# Neurofeedback-based functional near-infrared spectroscopy upregulates motor cortex activity in imagined motor tasks

**DOI:** 10.1117/1.NPh.4.2.021107

**Published:** 2017-06-23

**Authors:** Pawan Lapborisuth, Xian Zhang, Adam Noah, Joy Hirsch

**Affiliations:** a Columbia University, Fu Foundation School of Engineering and Applied Science, Department of Biomedical Engineering, New York, New York, United States; b Yale School of Medicine, Department of Psychiatry, New Haven, Connecticut, United States; c Yale School of Medicine, Department of Neuroscience, New Haven, Connecticut, United States; d Yale School of Medicine, Department of Comparative Medicine, New Haven, Connecticut, United States; e University College London, Department of Medical Physics and Biomedical Engineering, London, United Kingdom

**Keywords:** functional near-infrared spectroscopy, near-infrared spectroscopy, neurofeedback, biofeedback, brain–machine interface, imaginary motor tasks, neuroprosthetics

## Abstract

Neurofeedback is a method for using neural activity displayed on a computer to regulate one’s own brain function and has been shown to be a promising technique for training individuals to interact with brain–machine interface applications such as neuroprosthetic limbs. The goal of this study was to develop a user-friendly functional near-infrared spectroscopy (fNIRS)-based neurofeedback system to upregulate neural activity associated with motor imagery, which is frequently used in neuroprosthetic applications. We hypothesized that fNIRS neurofeedback would enhance activity in motor cortex during a motor imagery task. Twenty-two participants performed active and imaginary right-handed squeezing movements using an elastic ball while wearing a 98-channel fNIRS device. Neurofeedback traces representing localized cortical hemodynamic responses were graphically presented to participants in real time. Participants were instructed to observe this graphical representation and use the information to increase signal amplitude. Neural activity was compared during active and imaginary squeezing with and without neurofeedback. Active squeezing resulted in activity localized to the left premotor and supplementary motor cortex, and activity in the motor cortex was found to be modulated by neurofeedback. Activity in the motor cortex was also shown in the imaginary squeezing condition only in the presence of neurofeedback. These findings demonstrate that real-time fNIRS neurofeedback is a viable platform for brain–machine interface applications.

## 1 Introduction

Neurofeedback has recently been a topic of interest among engineers and neuroscientists due to potential benefits in clinical and commercial applications.^1^ Neurofeedback methods have been shown to enhance recovery of normal brain function in patients with brain injuries, especially in poststroke victims.^2^^,^^3^ It has also recently been shown to be a promising technique in developing brain–machine interface applications, such as neuroprosthetics and artificial vision.^4^^,^^5^

Most neurofeedback systems currently utilize electroencephalography (EEG) or functional magnetic resonance imaging (fMRI) to acquire the neural activity of the individual performing the task. Previous studies have demonstrated that EEG neurofeedback can be used to teach participants how to control cursor movements in one and two dimensions by modifying neural activity.^6^^–^^8^ However, a major obstacle in developing EEG brain–machine interface applications lies in the difficulty of localizing signal components associated with actual physiological movements.^9^ Prior neuroimaging studies have replicated the results of EEG neurofeedback by teaching participants to control cursor movements in multiple dimensions using fMRI neurofeedback systems based on the blood oxygenation level-dependent (BOLD) signal from the selected region of interest (ROI).^10^ Recent experiments have further demonstrated the use of fMRI neurofeedback for teaching participants to control the movement of robotic arms, a task directly related to the control of neuroprosthetics.^5^^,^^11^ Although these are encouraging outcomes, EEG’s poor spatial resolution and fMRI’s limited experimental environment are barriers to the use of neurofeedback for the development of brain–machine interface applications.

Functional near-infrared spectroscopy (fNIRS) is a neuroimaging modality that measures changes in optical densities and converts these to changes in hemoglobin concentrations as a proxy for neural activity. This relationship depends upon neurovascular coupling and may be influenced by the physiological state of the subject. However, fNIRS provides an alternative to EEG and fMRI as a noninvasive brain imaging technique that may be used for neurofeedback in natural conditions. Spatial resolution is improved in comparison to EEG and temporal resolution is enhanced in comparison to fMRI. Over the past decade, fNIRS has been used as a neuroimaging tool in various fields of studies, including psychiatry, psychology, and basic neuroscience research.^12^ Most notably, fNIRS has been applied to numerous language studies in human newborns and adults.^13^^,^^14^ Recently developed NIRS machines have a spatial resolution of ∼3  cm and a temporal resolution of 10 ms. Another major advantage of NIRS is that it allows participants to remain in a relatively natural environment while undergoing the training. Although the goal of this experiment is to understand early neural activity in untrained participants associated with neurofeedback, a long-term goal is to train participants to manipulate these signals. The ability to train in a natural environment is particularly important when learning to control a prosthetic limb. Such training is impossible within an MRI machine in which any motion >2  mm results in signal artifact.

Despite the benefits that fNIRS offers over EEG and fMRI, the potential use of fNIRS in neurofeedback is not well defined. Previous studies have shown that fNIRS neurofeedback can be used to increase cortical activation associated with motor imagery tasks when participants were trained over time as well as in comparison to sham feedback.^15^^,^^16^ Further questions concerning the development of fNIRS brain–machine interface applications, including identification of additional neural components associated with motor imagery and neurocircuitry associated with effectively incorporating neurofeedback to enhance performance, remain unanswered. The main goal of this study was to develop and test an fNIRS neurofeedback system that utilizes the large amplitude signal from oxyhemoglobin (OxyHb) changes in the motor cortex associated with upper limb motor tasks as the feedback signal for the participant while analyzing the neural effects of neurofeedback using the deoxyhemoglobin (deOxyHb) signal. We specifically investigated the potential of fNIRS feedback as a platform for brain–machine interface applications by determining changes in hemodynamic responses during motor imagery tasks associated with neural feedback without longitudinal training. We hypothesized that untrained participants would produce increased activity in the motor cortex in the presence of neurofeedback compared with no feedback, especially during motor imagery tasks.

## 2 Methods

### 2.1 Participants

A total of 22 individuals (14 female, mean age=24.5±7.8  years) participated in the experiment. All but two participants were right handed as determined by the Edinburgh Handedness Inventory.^17^ Written informed consent was obtained from all participants for participation in the study in accordance with guidelines approved by the Yale University Institutional Review Board (HIC #1501015178).

### 2.2 Experimental Design

**Fig. 1:**
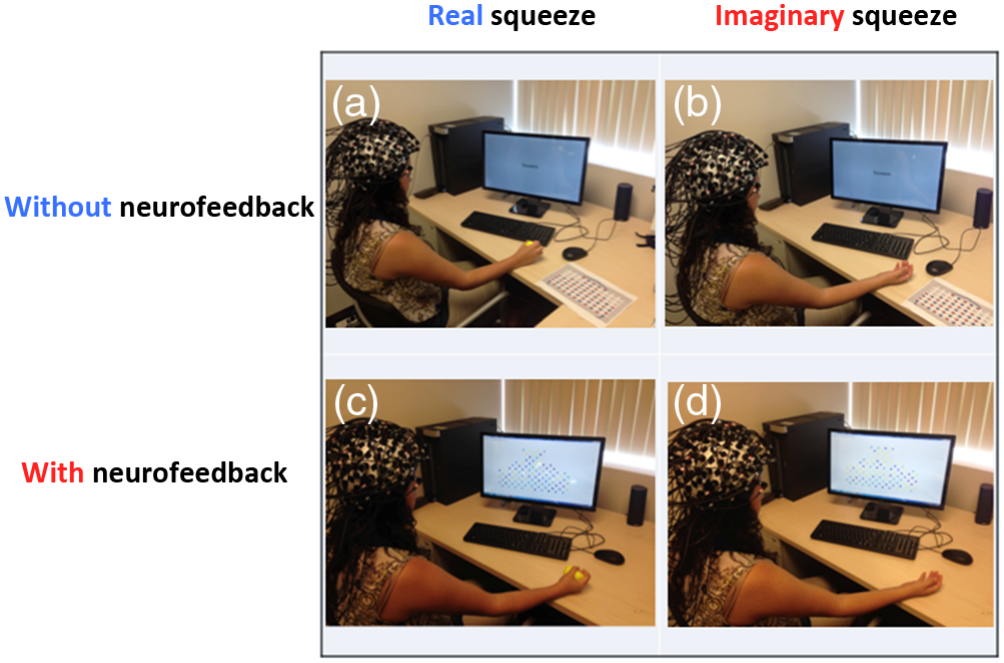
2×2 graphic demonstrating the conditions in the order in which participants performed the tasks (a) real squeezing without neurofeedback, (b) imaginary squeezing without neurofeedback, (c) real squeezing with neurofeedback, and (d) imaginary squeezing with neurofeedback.

Participants completed a series of active and imaginary hand-squeezing motor tasks using an elastic stress ball. During the active task, participants were asked to perform a rhythmic squeezing movement at a rate of approximately one squeeze per second during active blocks lasting 15 s. Rest periods of 15 s were interleaved with the active periods. The block design consisted of six repetitions for a total of 3 min. Participants repeated the same block design during the imaginary squeezing task, but they were asked to imagine themselves squeezing an elastic ball at a rate of approximately one squeeze per second. Participants were asked to perform both the real and the imaginary squeezing tasks under two conditions, with and without neurofeedback. Tasks were performed in the following order: real squeezing without neurofeedback, imaginary squeezing without neurofeedback, real squeezing with neurofeedback, and imaginary squeezing with neurofeedback (Fig. 1). Participants were instructed to perform the task sequence twice, completing the experiment with a total of eight runs, two runs per each condition.

### 2.3 fNIRS Neurofeedback System

**Fig. 2:**
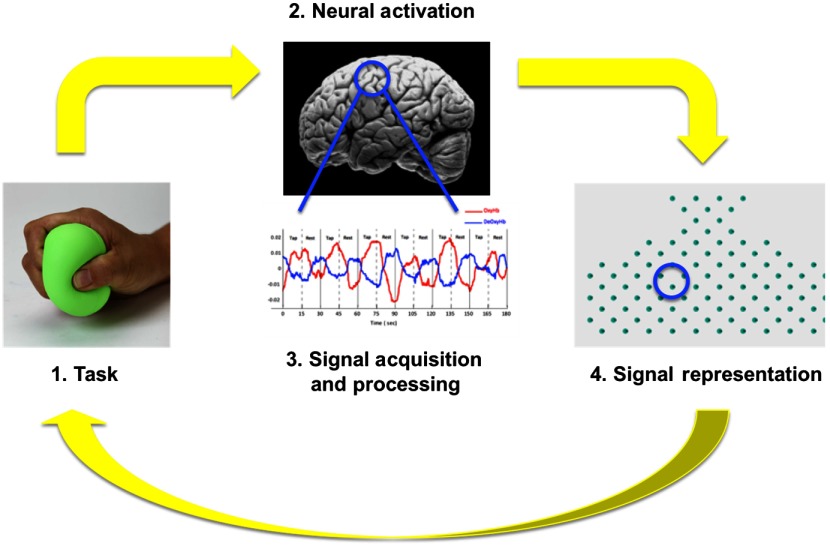
Overview of neurofeedback system: participants engage in the task, resulting in neural activation, which is acquired using the fNIRS system. The signal is processed and displayed to the participant, who then attempts to modify his/her behavior to change the representation of the signal.

**Fig. 3:**
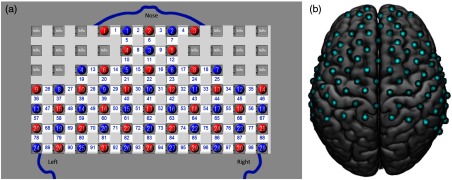
(a) NIRS optode channel diagram with emitters shown in red circles, detectors shown in blue circles, and channel numbers shown between emitters and detectors. For orientation, left and right hemispheres are labeled as well as the relative location of the nose. (b) Channel layout rendered on a standard template brain (dorsal view) shows coverage of frontal, temporal, and parietal lobes. Top of the figure is frontal; bottom of the figure is posterior. Hemispheres are labeled in (a).

An overview of the neurofeedback pathway used in this experiment is shown in Fig. 2. The major components of the fNIRS neurofeedback system consist of the fNIRS machine, the processing computer, and the screen on which feedback based on the OxyHb was displayed. Local changes in both the OxyHb and the deOxyHb were acquired on a LABNIRS fNIRS system (Shimadzu Corp., Kyoto, Japan). Thirty emitters and 29 detectors were secured in place 3 cm apart using a customized elastic cap. The channel layout along with the emitters and the detectors used in this experiment is shown in Fig. 3. The anatomical locations of optodes in relation to standard head landmarks (including inion; nasion; top center, Cz; left, T3; and right, T4) were determined for each participant using a Patriot 3-D Digitizer (Polhemus, Colchester, Vermont) and linear transform techniques as previously described.^18^^–^^21^ The Montreal Neurological Institute (MNI) coordinates for the channels were obtained using the NIRS_SPM software^17^ with MATLAB (Mathworks, Natick, Massachusetts), and the corresponding anatomical locations of each channel were determined by the Talairach atlas provided in SPM8.

**Fig. 4:**
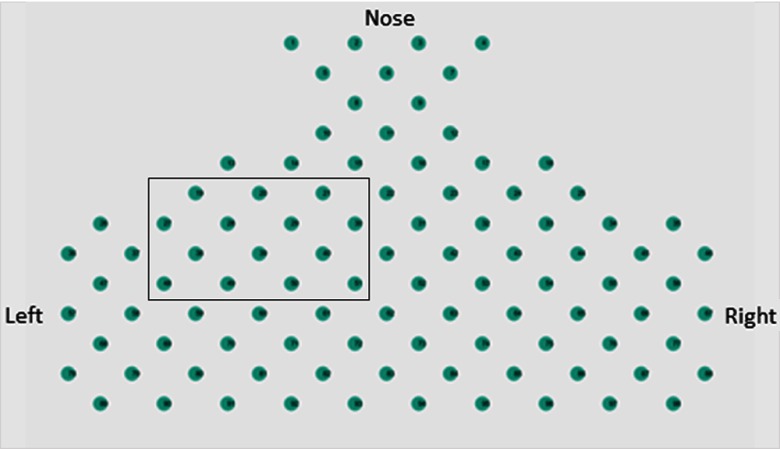
Signal display used to represent neurofeedback data to the participant, shown here at baseline. The color of the dots changed dynamically according to activity level. Yellow represented high activity, blue represented low activity, and green represented baseline activity. For orientation, left and right hemispheres are labeled as well as the relative location of the nose. The general ROI in the motor cortex is illustrated by the outlined rectangle in the figure.

Because of the relatively high amplitude response of OxyHb, this signal was used to provide visual representation during neurofeedback trails. Visual feedback was presented by determining the difference between the current sample and a linear trend of the previous 10 s of data. The presented data were normalized by dividing the current sample by the standard deviation of the previous 10 s of data. The detrended signal was used to generate the graphical image used for neurofeedback. Neurofeedback was displayed to each participant using a customized MATLAB graphic user interface (GUI) with colored dots representing the NIRS optode channel layout used in this experiment (Fig. 4). The layout and anatomical representation were explained to participants prior to the experiment. Participants were told that each dot on the GUI represented the neural activity level at a different location. Each colored dot represents a single channel. Yellow represented high activity, blue represented low activity, and green represented baseline activity. The color of the dots was updated each second depending upon the real-time activity level at each optode location using signal processing described above. Participants were instructed to focus on the group of channels covering the motor cortex contralateral to the hand used for squeezing tasks as shown in the black rectangle in Fig. 4.

### 2.4 Data Analysis

#### 2.4.1 Signal processing

Baseline drift was modeled and removed using a polynomial of the fourth degree,

$$P(t)=a_{0}+a_{1}t+a_{2}t^{2}+a_{3}t^{3}+a_{4}t^{4},$$

which was fitted to the raw fNIRS signals (MATLAB). Any channel without a signal due to insufficient optode contact with the scalp was identified by taking the root mean square of the raw data when the signal magnitude was more than 10 times the average signal and removed automatically.

#### 2.4.2 General linear model analysis

Previous NIRS studies have typically focused on analyses of neural activity as reflected by the OxyHb signal due to its large amplitude and higher signal-to-noise ratio.^16^ However, recent studies have demonstrated that the deOxyHb signal shows a higher correlation to the hemodynamic response measured through the BOLD signal using fMRI,^22^ may better represent neural activity, and contains less systemic artifact than the OxyHb signal.^23^^,^^24^ While we used the OxyHb signal to provide feedback to participants, analyses described here representing neural activity in response to either active or imagined tasks are based on deOxyHb signals. For reference, results from both OxyHb and deOxyHb signals before and after spatial filtering are presented in Fig. 6.

Each participant’s data sets were first reshaped into 3D volume images for the first-level general linear model (GLM) analysis using SPM8. DeOxyHb signals were compared between periods of activity and rest for each condition using GLM analysis function in the NIRS_SPM toolbox.^25^^,^^26^ For group data, the beta values (i.e., the amplitude of the deOxyHb signal) were normalized to standard MNI space using linear interpolation. The computational mask was subdivided into a total of 3,753 2×2×2  mm voxels that “tiled” the shell region covered by the 98 channels. Results were rendered onto a standard 3-D brain map, and GLM results were then compared across conditions between real and imaginary motor tasks and tasks performed with and without neurofeedback. Interpolated anatomical locations of peak voxel activity were identified using NIRS_SPM.^26^^,^^27^

#### 2.4.3 Small volume analysis

**Fig. 5:**
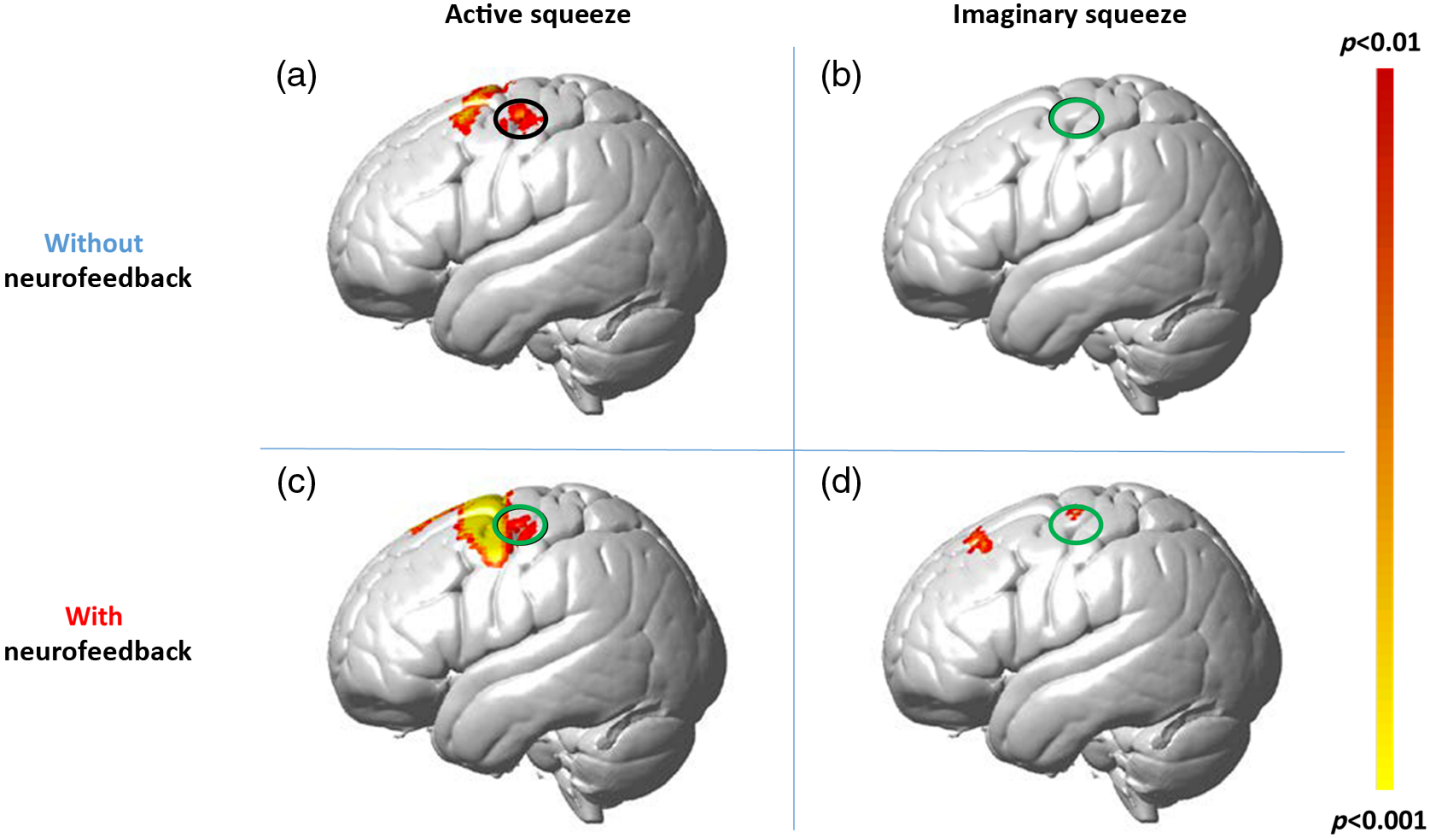
Factorial comparisons showing neural activity during active motor tasks (a, c) and imagined motor tasks (b, d) without neurofeedback (a, b) and with neurofeedback (c, d) (N=22, p<0.01). Only positive results from deOxyHb signals in the left hemisphere are shown. Results show increased activity in left premotor and supplementary motor cortex in the presence of neurofeedback for both active and imagined trials. Black oval outlines surround the ROI defined by the active squeezing without neurofeedback in the precentral gyrus. Green ovals show this ROI superimposed on the other conditions, showing no activity in the imaginary squeeze without neurofeedback condition and activity in both neurofeedback conditions within the ROI [family-wise error (FWE), p<0.05].

A small volume analysis was performed on voxelwise results using the functional cluster determined from right-handed ball squeezing in the contralateral motor area. The cluster of activity used to generate the functional mask is circled in black in Fig. 5(a). Signal processing related to neurofeedback was restricted within this small volume during right-handed ball squeezing with neurofeedback as well as during the two imagined task conditions (with and without neurofeedback).

## 3 Results

The results are shown in factorial design (Fig. 5). The left column represents the results of the active ball-squeezing task, whereas the right column shows the result of the imagined squeezing task. The top row displays group activation data rendered on a standard brain without neurofeedback. The bottom row shows activity from trials in the presence of neurofeedback. Figure 5(a) shows the result of active ball squeezing without neurofeedback: two clusters of activity located in the premotor, motor, and supplementary motor cortex. The posterior cluster has a peak MNI coordinate at (−52,14,6) (p<0.01, t=3.17, no. of voxels=128), and the anterior cluster has a peak MNI coordinate at (−12,0,72) (p<0.01, t=4.09, no. of voxels=568). No activity above threshold (p<0.05) was found in the left hemisphere for the imaginary motor task without neurofeedback [Fig. 5(b)].

Adding neurofeedback to the active squeezing task [Fig. 5(c)] resulted in a larger cluster of activity in the premotor and primary motor cortex with a peak MNI coordinate at (−34,4,62) (p<0.01, t=5.13, no. of voxels=2530). The addition of neurofeedback to the imaginary motor task resulted in one cluster in premotor cortex and another in the frontal eye fields. The premotor cluster has a peak MNI coordinate at (−40,−12,66) (p<0.01, t=3.31, no. of voxels=78), and the cluster in the frontal lobe has a peak MNI coordinate at (−34,26,50) (p<0.01, t=3.20, no. of voxels=74, see Table 1).

**Table 1:** GLM results from statistical parametric mapping (SPM) analysis (deOxyHb signals). Peak coordinates are based on the MNI system and (−) on the x-axis indicates left hemisphere. MNI coordinates were converted to Talairach coordinates to generate cluster labels including anatomical areas and the probability that the cluster is indicated in the labeled region (right column). The t value with the associated probability (p) indicates level of significance of the peak MNI coordinates. Number of voxels is an indicator of the size of the cluster. t, t value; p, p value; BA, Brodmann’s area.

Contrast (p<0.01)	Peak MNI coordinates	t	p	No. of voxels	BA	Anatomical area	Probability
Active squeezing without neurofeedback	(−52,14,56)	3.17	0.00232	128	6	Premotor and supplementary motor cortex	0.42
					3	Primary somatosensory cortex	0.22
					1	Primary somatosensory cortex	0.13
					2	Primary somatosensory cortex	0.12
					4	Primary motor cortex	1.00
	(−12,0,72)	4.09	0.00026	568	6	Premotor and supplementary motor cortex	
	(58,−48,30)	3.41	0.00131	89	40	Supramarginal gyrus part of Wernicke’s area	0.84
	(24, 26, 58)	3.26	0.00187	112	6	Premotor and supplementary motor cortex	0.52
					8	Frontal eye fields	0.48
Imaginary squeezing without neurofeedback	(70,−26,2)	3.16	0.00238	57	21	Middle temporal gyrus	0.39
					22	Superior temporal gyrus	0.35
					42	Primary and auditory association cortex	0.25
	(68,−36,28)	3.86	0.00046	101	40	Supramarginal gyrus part of Wernicke’s area	0.72
					22	Superior temporal gyrus	0.16
	(56, 22, 30)	3.62	0.0008	59	9	Dorsolateral prefrontal cortex	0.51
					46	Dorsolateral prefrontal cortex	0.23
					45	Pars triangularis Broca’s area	0.20
Active squeezing with neurofeedback	(−34,4,62)	5.13	0.00002	2530	6	Premotor and supplementary motor cortex	0.96
	(62,−48,38)	3.79	0.00053	128	40	Supramarginal gyrus part of Wernicke’s area	0.98
Imaginary squeezing with neurofeedback	(−40,−12,66)	3.31	0.00167	78	6	Premotor and supplementary motor cortex	0.74
					3	Primary somatosensory cortex	0.17
	(−34,26,50)	3.20	0.00213	74	8	Frontal eye fields	0.89
	(20, 46, 50)	3.78	0.00055	87	8	Frontal eye fields	0.79
					9	Dorsolateral prefrontal cortex	0.21

### 3.1 Small Volume Results

Results for imagined ball squeezing without neurofeedback [Fig. 5(b)] showed no significant clusters of activity in the functionally defined small volume in the motor cortex determined by active ball squeezing. The green circle is shown for reference and represents the same area in Fig. 5(a) outlining the functional activity during ball squeezing in the motor cortex. The cluster of activity in this same small volume for the ball squeezing with neurofeedback [Fig. 5(c)] and imaginary condition with neurofeedback [Fig. 5(d)] indicated the effects of neurofeedback (p<0.05; FWE correction).

## 4 Discussion

The results of this study demonstrate the utility of an fNIRS-based neurofeedback system for upregulating motor cortex activity with minimal training. Specifically, participants showed increased activity in the motor cortex during neurofeedback for both active and imagined motor tasks compared with the same tasks in the absence of neurofeedback. Additional bilateral activity was seen in the prefrontal cortex during the imagined task with neurofeedback. These results also suggest that fNIRS neurofeedback can increase or regulate brain activity with minimal training.

### 4.1 Use of OxyHb Signal for Neurofeedback

OxyHb and deOxyHb hemodynamic signals represent relative changes in blood oxygen levels^28^ reflecting underlying neural activity, similar to magnetic resonance measured in fMRI.^12^^,^^29^^,^^30^ The OxyHb signal recorded using fNIRS includes additional components originating from systemic effects, such as blood flow, blood pressure, and respiration compared with the deOxyHb signal^12^^,^^23^^,^^29^^,^^30^ and has an increased spatial distribution compared with the deOxyHb signal.^24^

Despite the susceptibility of the OxyHb signal to systemic artifacts, localized increases in OxyHb can be used as a visual proxy for cortical processing in the motor cortex and other areas where the signals are very large. It is difficult to differentiate cortical responses from systemic effects, but for presentation of the biofeedback, this is less important as long as a signal is present and can be seen by the participant. DeOxyHb signals are often smaller in amplitude and contain noise that requires modeling to determine activity that follows a hemodynamic response function even for studies that focus on group level activity. In this study, we took advantage of large amplitude changes in the OxyHb signal to provide single participants with graphical representations of localized individual neural activity, from which they could learn to manipulate through focused attention or other mechanisms. We found that activity recorded from a single trial from a single participant was enough to provide a signal that could be presented to an individual as a source of neurofeedback. Responses localized to the contralateral motor cortex were large enough in amplitude that they could be seen by participants in real time using the neurofeedback GUI.

**Fig. 6:**
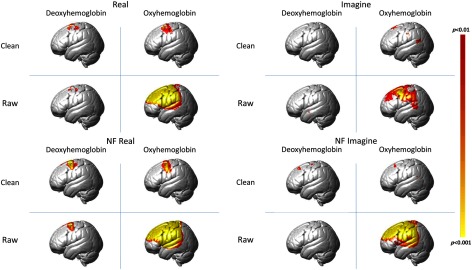
Renderings showing hemodynamic activity for both oxyHB and deoxyHB. Only left hemisphere results are shown. Real ball squeezing is shown on the left and imaginary squeezing is shown on the right. Raw signals are shown and compared with spatially filtered (clean) signals. NF refers to runs with neurofeedback.

While the OxyHb signal was suitable for neurofeedback visualization (as demonstrated in this experiment), the deOxyHb signal was used to measure activity associated with the active and imagined motor tasks in this study.^24^ Results from both signals are shown in Fig. 6. Results indicate that the left precentral gyrus ROI, shown to be active during right hand movements,^31^^,^^32^ displays increased activity in the presence of neurofeedback during real and imaginary motor tasks. These findings are consistent with other functional imaging studies using fMRI and EEG that have demonstrated similar increases in cortical activity during real and imaginary motor movements.^33^^,^^34^

### 4.2 Potential Mechanism of Upregulated Activity

In addition to shared neural components between the real and imaginary motor tasks, results also highlight additional recruitment of neural processes during imaginary motor tasks with neurofeedback. Activity in the bilateral middle frontal gyri, which is only present in the imagined task with neurofeedback, may suggest the incorporation of executive control mechanisms originating in the prefrontal cortex. Previous neurofeedback studies have also found bilateral activity in the middle frontal gyri associated with goal completion, anticipation, goal selection, planning, and initiation of activity, self-regulation, monitoring, and use of feedback.^35^

Imaginary motor tasks may require additional spatial processing and working memory abilities relative to real motor tasks. This finding suggests that additional attentional effort and maintenance may be necessary to produce activity in the imagined condition. Such activity may represent neurofeedback-based facilitation of information transfers between the two brain regions. These results lend additional support to prior studies demonstrating altered functional connectivity during imagined motor tasks with real-time fMRI neurofeedback.^36^^,^^37^

## 5 Limitations

Although very little training was necessary to increase motor activity in the presence of neurofeedback provided by the OxyHb signal in this experiment, further investigation is needed to study the longitudinal effects of fNIRS neurofeedback in changing or shaping neural activity patterns and functional connectivity. The current study showed that naïve participants could successfully use fNIRS neurofeedback to increase activation in brain regions associated with motor imagery and executive function, and further suggests that fNIRS neurofeedback has potential for training participants to control brain activity associated with more complex tasks.

The active squeezing condition was always first, followed by imaginary squeezing, followed by active squeezing with neurofeedback, and then imaginary squeezing with neurofeedback. This sequence was repeated twice for each subject. To maximize the ability of the subjects to use neurofeedback without training, the neurofeedback runs were always presented after the nonneurofeedback runs. However, if there was an effect of habituation as subjects performed the tasks, we would expect that later runs would show less activity. Nonetheless, neurofeedback runs showed increased activity compared with nonneurofeedback runs, indicating that if there was an effect of habituation, the activity for neurofeedback runs could be even greater than shown here. The imaginary squeezing runs also always followed the real squeezing runs. Decreased activity due to habituation may explain the lack of results during the imaginary squeezing without neurofeedback condition.

Another limitation of the current study was the presence of physiological noise in the real-time OxyHb signal presented to the participants. Future studies incorporating a real-time global mean removal algorithm^24^ should be conducted in order to determine the effects of a more localized and specific neurofeedback on the participant’s neural circuitry associated with motor imagery tasks.

## 6 Conclusion

The current study demonstrates the potential of fNIRS as a platform for the development of real-time neurofeedback systems for brain–machine interface applications such as neuroprosthetics. The results of the study show that an fNIRS neurofeedback system can be used to increase neural activity associated with simple motor imagery with minimal training. Although similar studies have been performed using EEG and fMRI, our results reveal additional details regarding the neurocircuitry associated with motor imagery and neurofeedback. With an improved spatial resolution relative to EEG and a more natural experimental setting than fMRI, fNIRS may be uniquely suitable for clinical applications. Further studies are necessary to investigate how fNIRS neurofeedback modulates brain activity during complex tasks and how brain activity changes after extended training sessions.
